# Evaluation of Supplemental Benefits Across Medicare Advantage Plans and Beneficiary Demographic Characteristics, 2019 to 2022

**DOI:** 10.1001/jamanetworkopen.2022.33020

**Published:** 2022-09-23

**Authors:** Neil P. Rowen, Lucas Stewart, Robert S. Saunders

**Affiliations:** 1 Duke-Margolis Center for Health Policy, Duke University, Durham, North Carolina; 2 Duke-Margolis Center for Health Policy, Duke University, Washington, DC

## Abstract

This cross-sectional study evaluates how policy changes for Medicare Advantage plans affected supplemental benefit availability across geographic social vulnerability, membership diversity, and rebates from 2019 to 2022.

## Introduction

The US Centers for Medicare and Medicaid Services (CMS) details 3 avenues that Medicare Advantage (MA) plans have to address social determinants of health through supplemental benefits. In 2018, primarily health-related (PHR) benefits expanded to include adult day health services, home-based palliative care, and in-home support services, among others.^1^ In 2020, the Creating High-Quality Results and Outcomes Necessary to Improve Chronic Care Act authorized Special Supplemental Benefits for the Chronically Ill (SSBCI), allowing plans to offer benefits such as food, transportation, pest control, and structural home modifications.^2^ Finally, flexibilities to address COVID-19 enabled plans to offer face masks, care packages, and reduced cost-sharing starting in 2021.

Supplemental benefits hold promise for addressing social needs as overall MA enrollment (28.5 million) increases alongside enrollment in plans offering PHR (3.3 million) or SSBCI (2.7 million) benefits.^3^ We examined how CMS policy changes affected supplemental benefit availability across geographic social vulnerability, membership diversity (in terms of race and preferred language), and rebates from 2019 to 2022. Our focus on US demographic and resource differences adds to prior work detailing supplemental benefit availability and enrollment.^4,5^

## Methods

Because deidentified publicly available data were used, Duke University institutional review board policy classified this cross-sectional study as not human participants research, which did not require institutional review board approval and waived informed consent. The study followed the STROBE reporting guideline.

Data sources included CMS MA plan benefit package, enrollment, Healthcare Effectiveness Data Information System (HEDIS), and plan payment files. Social Vulnerability Index (SVI) data were from the US Centers for Disease Control and Prevention. We excluded plans that offered benefits before CMS policy changes or followed different regulations (eg, Dual Eligible Special Needs, Financial Alignment Initiative, Employer Group Waiver, Program of All-Inclusive Care for the Elderly, Medical Savings Accounts, Cost, Part B only, and Prescription Drug plans). MA HEDIS data for race and preferred language were self-reported. The eAppendix in the Supplement provides additional details on data sources and methods. We conducted 2-tailed *t* tests, with significance at *P* < .05. Mean values are presented with 95% CIs. Statistical analysis was performed with R version 4.1.3 (R Project for Statistical Computing).

## Results

**Figure 1.**
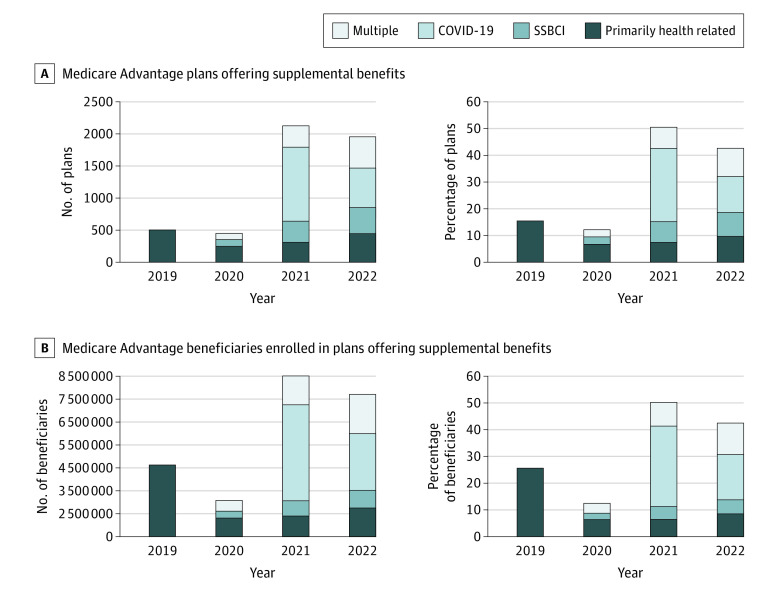
Types of Supplemental Benefits Offered by Medicare Advantage Plans and Beneficiaries Enrolled The 3 individual categories (primarily health related, Special Supplemental Benefits for the Chronically Ill [SSBCI], and COVID-19) indicate plans only offering that benefit type, whereas the multiple category indicates a plan offering any combination of the 3 types.

Results for 2022 showed that 1959 of 4583 MA plans (42.7%) provided PHR, SSBCI, or COVID-19 benefits, with 42.6% of beneficiaries enrolled. Figure 1 demonstrates that 1174 plans (25.6%) offered SSBCI, PHR, or both benefit types, with 20.6% of beneficiaries enrolled; 785 plans (17.1%) offered COVID-19 benefits (alone or in combination), with 17.1% of beneficiaries enrolled. Of plans that offered multiple supplemental benefits, 318 (6.9%) offered both PHR and SSBCI, with 6.8% of beneficiaries enrolled.

**Figure 2.**
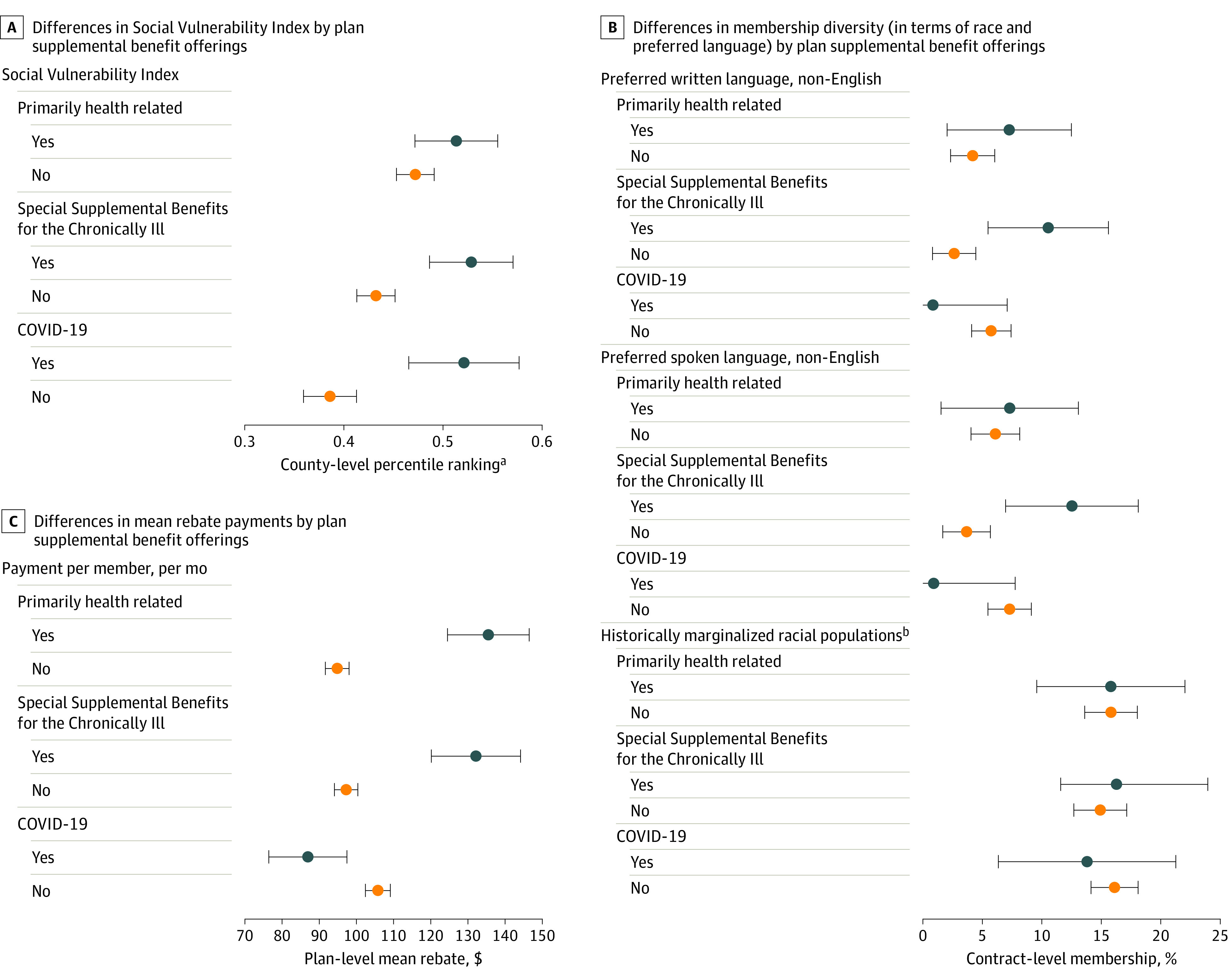
Differences in Social Vulnerability Index Scores by Medicare Advantage Plan Supplemental Benefit Offerings Dot and error bar values represent means (95% CIs). ^a^Higher rankings indicate more social vulnerability. ^b^These values were obtained by summing the percentages in Medicare Advantage data on membership diversity for historically marginalized racial populations (ie, American Indian or Alaska Native, Asian, Black or African American, or Native Hawaiian or Other Pacific Islander). Details are provided in the eAppendix in the Supplement.

Figure 2 illustrates differences across plans based on whether they offered supplemental benefits. Plans that offered benefits were available in US counties with higher vs lower mean SVI scores (PHR: 0.51 [0.47-0.56] vs 0.47 [0.45-0.49]; SSBCI, 0.53 [0.49-0.57] vs 0.43 [0.41-0.45]; and COVID-19: 0.52 [0.47-0.58] vs 0.39 [0.36-0.41]; all *P* < .001). Plans that offered SSBCI benefits had greater mean percentages of beneficiaries who reported that English was not their preferred spoken (12.5% [7.0%-18.1%] vs 3.7% [1.7%-5.7%]) or written (10.5% [5.5%-15.6%] vs 2.6% [0.8%-4.5%]) language (both *P* < .001). Mean rebates were higher among plans that offered PHR ($135.49 [$124.50-$146.48] vs $94.84 [$91.65-$98.03]) or SSBCI ($132.16 [$120.16-$144.17] vs $97.25 [$94.10-$100.39]) benefits (both *P* < .001) vs those that did not.

## Discussion

Increasingly offered supplemental benefits give beneficiaries access to choose health plans that can address their unmet needs. In this cross-sectional study, we observed that MA plans offering SSBCI benefits were available in areas with higher social vulnerability and greater membership diversity.

Study limitations included incomplete data for race and preferred language and possible undercounting of benefits before 2020, given that benefit naming conventions were not standardized. Plans differ in aligning benefits across products, and wider benefit uptake may require additional evidence of return on investment.^6^ CMS recently sought public feedback on improving MA, and these findings have implications for their health equity efforts such as the proposed Health Equity Index for the Star Ratings program.

## Notes

Supplement. **eAppendix.** Data Sources and Methods
