# Supplementary material for: Evaluation of Supplemental Benefits Across Medicare Advantage Plans and Beneficiary Demographic Characteristics, 2019 to 2022
Source: JAMA Netw Open. 2022 Sep 23;5(9):e2233020. doi: 10.1001/jamanetworkopen.2022.33020 (PMC9508655; doi:10.1001/jamanetworkopen.2022.33020)
Supplement: Supplement. — eAppendix. Data Sources and Methods [file jamanetwopen-e2233020-s001.pdf]

Supplementary Online Content

Rowen NP, Stewart L, Saunders RS. Evaluation of supplemental benefits across Medicare Advantage plans and beneficiary demographic characteristics, 2019 to 2022. *JAMA Netw Open*. 2022;5(9):e2233020. doi:10.1001/jamanetworkopen.2022.33020

eAppendix. Data Sources and Methods

This supplementary material has been provided by the authors to give readers additional information about their work.

© 2022 Rowen NP et al. *JAMA Network Open*.

eAppendix. Data Sources and Methods

Disparities Analysis Variables

We used the Centers for Disease Control and Prevention Social Vulnerability Index (SVI) data. We defined other key variables, like race, language, and rebates, as follows. Race, Ethnicity, and Preferred Language data are based on the Medicare Advantage Healthcare Effectiveness Data and Information Set's (HEDIS) Race/Ethnicity Diversity of Membership (RDM) and Language Diversity of Membership (LDM) measures. The most recent year both measures were reported is 2019. Our person of color measure was created by summing the percentages reported for the RDM measure that were not white regardless of ethnicity. That is, the percentage of membership that is either Black or African American, Asian, American-Indian and Alaska Native, or Native Hawaiian or Other Pacific Islanders across ethnicity status. Declined or Unknown race was treated as a separate group during analysis. The MA Dictionary in each PUF HEDIS file has variable names and descriptions used by CMS. Rebates represent average per-member, per-month payments from Medicare Advantage Plan Payment data for 2019, the most recent year available.

Plan and Enrollment Numbers

We used MA plan benefit package and contract/plan/state/county-level enrollment data from CMS for our plan and enrollment analyses. Our results exclude exclude Dual Eligible Special Needs Plans, Financial Alignment Initiative Medicare-Medicaid plans, Employer Group Waiver Plans, Program of All-Inclusive Care for the Elderly plans, Medical Savings Accounts, Cost, Part B only, and Prescription Drug plans. Beneficiary numbers are for January of indicated year. COVID-19 benefits are only available from 2021 onward. SSBCI are only available from 2020 onward. The underlying benefits for each main supplemental benefit category are listed below. The benefit groupings are based on CMS policy changes and regulatory actions.

Supplemental Benefit Category	Supplemental Benefit
Primarily health-related	Adult day health services
	Home-based palliative care
	In-home support services
	Caregiver support
	Therapeutic massage (previously non-opioid pain management)
Special Supplemental Benefits for the Chronically Ill	General supports for living
	Food & produce
	Meals (beyond limited basis)
	Pest control
	Transportation for non-medical needs

© 2022 Rowen NP et al. *JAMA Network Open*.

	Indoor Air Quality Equipment and Services
	Social Needs Benefit
	Complementary Therapies
	Services Supporting Self-Direction
	Structural Home Modifications
COVID-19<br>(search text strings for any indication of these terms)	B13d: Personal Protective Equipment
	B13e: COVID-19; COVID-19 care package; COVID-19 reduced cost share; waived hospital payment for COVID treatment
	B13f: Covid 19; COVID-19; COVID-19 emergency cost share reduction; COVID-19 testing and specified testing-related services; face masks; Healthy Food via Primary Care Orders during COVID-19; Public Health Emergency; Public Health Emergency Package

© 2022 Rowen NP et al. *JAMA Network Open*.
